# A program of nurse algorithm-guided care for adult patients with acute minor illnesses in primary care

**DOI:** 10.1186/1471-2296-14-61

**Published:** 2013-05-16

**Authors:** Núria Fabrellas, Carmen Sánchez, Eulàlia Juvé, Eva Aurin, Dolors Monserrat, Esther Casanovas, Magali Urrea

**Affiliations:** 1 School of Nursing, University of Barcelona, Institut d’Investigació Biomèdica de Bellvitge (IDIBELL), Campus de Ciències de la Salut Bellvitge, Feixa Llarga s/n Pavello de Govern, 3a planta, L’Hospitalet de Llobregat, Catalunya, 08907, Spain; 2 SAP Mataró, Institut Català de la Salut, Mataró, Catalunya, Spain; 3 Institut Català de la Salut, Barcelona, Catalunya, Spain; 4 EAP Premià, Institut Català de la Salut, Premià de Mar, Catalunya, Spain; 5 EAP Pineda de Mar, Institut Català de la Salut, Pineda de Mar, Catalunya, Spain

**Keywords:** Nurse case management, Acute diseases/nursing, Nurse practitioner, Primary health care

## Abstract

**Background:**

Attention to patients with acute minor-illnesses requesting same-day consultation represents a major burden in primary care. The workload is assumed by general practitioners in many countries. A number of reports suggest that care to these patients may be provided, at in least in part, by nurses. However, there is scarce information with respect to the applicability of a program of nurse management for adult patients with acute minor-illnesses in large areas. The aim of this study is to assess the effectiveness of a program of nurse algorithm-guided care for adult patients with acute minor illnesses requesting same-day consultation in primary care in a largely populated area.

**Methods:**

A cross-sectional study of all adult patients seeking same day consultation for 16 common acute minor illnesses in a large geographical area with 284 primary care practices. Patients were included in a program of nurse case management using management algorithms. The main outcome measure was case resolution, defined as completion of the algorithm by the nurse without need of referral of the patient to the general practitioner. The secondary outcome measure was return to consultation, defined as requirement of new consultation for the same reason as the first one, in primary care within a 7-day period.

**Results:**

During a two year period (April 2009-April 2011), a total of 1,209,669 consultations were performed in the program. Case resolution was achieved by nurses in 62.5% of consultations. The remaining cases were referred to a general practitioner. Resolution rates ranged from 94.2% in patients with burns to 42% in patients with upper respiratory symptoms. None of the 16 minor illnesses had a resolution rate below 40%. Return to consultation during a 7-day period was low, only 4.6%.

**Conclusions:**

A program of algorithms-guided care is effective for nurse case management of patients requesting same day consultation for minor illnesses in primary care.

## Background

In the current context of rapidly growing healthcare demands there is generalized interest for an increasing role of nurses in primary care [1]. The USA Institute of Medicine has called for broadening nurses’ scope of practice in primary care [2]. Likewise, in the UK, the government has issued a policy that expands the role of nurses in the community practice [3]. Management of patients with acute minor illnesses is an exemplary case to be considered. In many countries, the healthcare to these patients is delivered on a same day basis (that is, the same day the consultation occurs) by general practitioners (GPs) that have to find a spot in their busy agendas to visit these patients. There is a large body of evidence from randomized studies and systematic reviews indicating that nurses can provide these patients with care of similar quality to that provided by GPs with a similar or even better patient satisfaction [4-11]. However, despite this available evidence, care of patients with acute minor illnesses is still, by and large, delivered by GPs in most countries with little or no role for nurses.

The main problems preventing an expansion of the role of nurses in primary care are the disparity in the levels of nursing education in different countries and some regulatory barriers, particularly drug prescription. In Spain, the education of registered nurses (RN) is at the graduate level but they can only prescribe certain drugs under the supervision of the GP. In order to expand the role of RN and still adhering with the legislation, the Catalonian Institute of Health developed a program of nurse care for patients with acute minor illnesses that uses management algorithms, which are included in the computerized health record system. This study assessed the effectiveness of this program over a two-year period in a highly populated area with approximately 6 million inhabitants.

## Methods

### Design of the study

This is a cross-sectional study with retrospective analysis of data from patients attended in a program of nurse algorithm-guided care for adults with acute minor illnesses in primary care. Data were collected prospectively in the electronic medical record at the time of patient consultation.

### Study population

Adult patients requesting consultation without appointment for 16 different acute minor illnesses over a two year period (April 2009-April 2011) were included in the study. The analyzed geographical area includes 284 primary care practices attending a population of approximately 6 million inhabitants in Catalonia, northeast of Spain. Consecutive sampling technique was used to include cases in the study. All subjects older than 14 years requesting same day consultation for any of 16 acute minor illnesses (see later) were included in the study.

### Program of nurse algorithm-guided care for patients with acute minor illnesses

The program was made by a group of expert nurses and GPs and consisted of a general protocol and 16 management algorithms of very common acute minor illnesses in adults seen in primary care [12]. The 16 acute minor illnesses included in the program were the following: skin injury, upper respiratory symptoms, sore throat, lower urinary symptoms, acute diarrhea, low back pain, increased arterial pressure, pink eye, burns, tooth pain, twisted ankle, emergency contraception, anxiety attacks, skin fold dermatitis, flu, and nose bleeding. Management algorithms were made following the most recent available evidence for each of the 16 conditions. Training sessions were performed in all practices before the implementation of the program. Once the program started, all patients seeking same day consultation were initially seen by a nurse. In all practices, there was one nurse dealing with these patients 5 days per week. All nurses of each practice participated in the program in turns. The general protocol included the following steps: 1) assessment of main signs and symptoms; 2) recognition of previous health problems and their treatments; 3) identification of the main reason for consultation. If the patient’s condition was included in one of the 16 minor illnesses the corresponding predefined management algorithm was applied. Algorithms included signs of alarm to identify patients with a potentially severe condition requiring consultation with a GP. If signs of alarm were not present, the nurse completed the algorithm that in some cases required drug prescription. If signs of alarm were detected, the nurse referred the patient to the GP, who was always available for a same day visit. The protocol and management algorithms were included in the computerized medical system so that all steps could be followed easily at the time of writing in patients’ electronic medical records. As an example, the management algorithms of the 4 most common minor illnesses are shown in Tables 1, 2, 3 and 4.

**Table 1:** Nurse treatment protocol for patients with skin injury

	
**Signs of alarm**	Involvement of head, joints, nerves and/or tendons
	Profuse hemorrhage
	Report to the police, if violence was involved
**Treatment approach**	**No signs of infection or bites**
	1. Cleansing and disinfection with clorhexidine
	2. Moist environment
	3. Suture, if needed (open injuries of less than 6 hours).
	4. Haemostasis, if needed.
	5. If pain, paracetamol 500 mg tid, as needed
	6. Tetanus prophylaxis, if needed.
	**Signs of infection or bite injury**
	1. Cleansing and disinfection with clorhexidine.
	2. Moist environment
	3. No suture
	4. Haemostasis, if needed
	5. If pain, paracetamol 500/1,000 mg qid, as needed.
	6. Tetanus prophylaxis, if needed. In bites, consider rabies prophylaxis
	7. Amoxycillin-clavulanic acid 500/125 mg tid for 8 days. Erithromycin 500 mg qid for 8 days in patients with known allergy to penicillin.

**Table 2:** Nurse treatment protocols for patients with upper respiratory symptoms

	
**Signs of alarm**	Asthma or chronic pulmonary obstructive disease
	Shortness of breath
	Immunosuppressive therapy
	Temperature >40°C
	Fever for more than 3 days
	Symptoms for more than 14 days
	Abnormal lung auscultation
	Pregnancy or lactation
	Decompensated cardiac disease
	Diabetes mellitus not well controlled
	Recent hospitalization
	Severe ear pain
	Severe headache
	Chest pain
	Severe weakness
**Treatment approach**	1. In patients with runny nose, cough with sputum, fatigue and/or body aches, paracetamol 500/1,000 mg/day tid or qid, plus frequent fluid intake.
	2. In patients with cough without sputum, dextrometorphan 15–30 mg tid or qid or codeine 10–20 mg every 4–5 hours (if patient does not take monoamino oxidase inhibitors).

**Table 3:** Nurse treatment protocol for patients with sore throat

	
**Signs of alarm**	Symptoms for more than 7 days
	Temperature >40°C or >38°C for more than 3 days
	Presence of asthma or chronic pulmonary obstructive disease
	Shortness of breath
	Abnormal lung auscultation
	Cardiac disease
	Diabetes mellitus
	Ear pain
	Chest pain
	Lesions in the mouth or pharyngeal cavity or pharyngeal deformity suggestive of peritonsilar abscess
	Lack of improvement in patients with previous treatment
	Altered general condition
	Local lymph nodes without pharyngeal exudate
	Pregnancy or lactation
	Anticoagulation therapy
	Immunosuppressive therapy
**Treatment approach**	1. Hygiene recommendations: increased fluid intake, no smoking, warm lemon water gargles, refrain from excess of carbohydrates.
	2. Paracetamol 500/1,000 mg tid or ibuprofen 400/600 mg tid. In patients with intolerance to NSAIDs, renal or cardiac failure, hypertension, ulcer disease, age >65 yr or allergy to NSAIDs, paracetamol should be given.
	3. If fever and pharyngeal exudates are present penicil.lin or fenoximethylpenicillin 500 mg bid for 8–10 days or amoxicillin 500 mg tid for 7 days. Erithromycin 500 mg qid for 8–10 days in patients with known allergy to penicillin.

**Table 4:** Nurse protocol for patients with lower urinary symptoms

	
**Signs of alarm**	Symptoms for more than 7 days
	Fever and/or chills
	Pregnancy or lactation
	Male sex
	Age greater than 65
	Diabetes mellitus
	Recurrent urinary tract infection (≥ 2 infections within a 6-month period)
	Recent urinary tract infection (less than 2 weeks)
	Pyelonephritis within one year period
	Bladder catheter
	Vaginal discharge
	Nausea and/or vomiting
	Abdominal pain
	Known abnormalities in urinary tract
	Gross hematuria
	Immunosuppressive therapy
**Treatment approach**	1. Amoxycillin-clavulanic acid 500/125 mg tid for 5 days or norfloxacin 400 mg bid for 3 days. Norfloxacin prescribed to patients with known allergy to penicillin.
	2. Hygiene recommendations: cleanliness of the genital area, frequent bladder voiding, cotton-crotch underwear.
	3. Return to consultation if signs of alarm develop (chills/fever, red urine, abdominal or lumbar pain…)

### Data collection and analysis

Data from all consecutive patients seen in the program during the 2-yr period of the study were downloaded directly from the computerized electronic health record system using specific software and incorporated into a database for analysis.

The main outcome measure of the study was case resolution, defined as completion of the management algorithm without the need of referral of the patient to the GP. The secondary outcome measure was return to consultation, defined as requirement of new consultation for the same reason as the first one in primary care, either with a nurse or GP, within a 7-day period. Comparison of frequencies of the different outcomes was performed with the chi-square test. The analysis was performed using the SPSS 15 for Windows (SPSS Inc, Chicago, IL).

### Ethical approval

The study was approved by the Institut d’Investigació en Atenció Primària (IDIAP Jordi Gol) and followed the current regulations for data confidentiality.

## Results

During a two-year period, more than 1,200,000 same-day consultations corresponding to 16 predefined acute minor illnesses in adults were performed by nurses under the current program using management algorithms in primary care. This corresponds to an average of approximately 50,000 consultations per month and 2,400 consultations per working day.

The 16 minor illnesses and their corresponding frequencies are shown in Table 5. The most common were skin injury, upper respiratory symptoms, sore throat, lower urinary symptoms, acute diarrhea, and low back pain. Each of these 6 minor illnesses had a frequency greater than 5% (range 23.6 to 6.3) and all 6 together accounted for more than three quarters of all consultations (77.5%). The less common minor illnesses were nose bleeding, flu, skin fold dermatitis, and anxiety attacks, with a frequency lower than 2% each. The remaining conditions, increased arterial pressure, pink eye, burns, tooth pain, twisted ankle, and emergency contraception, had individual frequencies between 2 and 5%. In the second year the number of consultations increased by 10.3% with respect to the first year (Table 5).

**Table 5:** Frequency of the 16 minor illnesses during the 2-year period

	**Frequency**		
**Category**	**First year**	**Second year**	**Total**
Skin injury	130,988 (22.8)	154,924 (24.4)	285,912 (23.6)
Upper respiratory symptoms	118,470 (20.6)	109,293 (17.2)	227,763 (18.8)
Sore throat	67,550 (11.7)	71,386 (11.3)	138,936 (11.5)
Lower urinary symptoms	49,996 (8.7)	69,126 (10.9)	119,122 (9.9)
Acute diarrhea	42,431 (7.4)	47,708 (7.5)	90,139 (7.5)
Low back pain	35,059 (6.1)	41,033 (6.5)	76,092 (6.3)
Increased arterial pressure	23,632 (4.1)	29,344 (4.6)	52,976 (4.4)
Pink eye	21,485 (3.7)	24,946 (3.9)	46,431 (3.8)
Burns	18,540 (3.2)	19,468 (3.1)	38,008 (3.1)
Tooth pain	16,302 (2.8)	19,319 (3.0)	35,621 (2.9)
Twisted ankle	12,789 (2.2)	13,427 (2.5)	26,216 (2.2)
Emergency contraception	13,652 (2.4)	10,974 (1.7)	24,626 (2.0)
Anxiety attacks	10,125 (1.8)	11,720 (1.8)	21,845 (1.8)
Skin fold dermatitis	5,794 (1.0)	5,676 (0.9)	11,470 (1.0)
Flu	5,927 (1.0)	3,635 (0.6)	9,562 (0.8)
Nose bleeding	2,448 (0.4)	2,502 (0.4)	4,950 (0.4)
**Total**	**575,189**	**634,480**	**1,209,669**

Numbers in brackets are percentages of the total number of patients during each period.

Overall, case resolution was achieved in a high proportion of cases (62.5%). The remaining 37.5% of cases were not solved by the nurse and had to be referred to the GP. The frequency of case resolution over the two-year period varied widely among the different minor illnesses (Table 6). Resolution rates were very high (over 90%) in conditions such as burns, emergency contraception, or skin injury. In other minor illnesses, such as skin fold dermatitis, increased arterial pressure, twisted ankle, nose bleeding, acute diarrhea, tooth pain, anxiety attacks, and low back pain, resolution rates ranged between 50 and 70%. The lowest resolution rates (below 50%) were found in upper respiratory symptoms, pink eye, lower urinary symptoms, sore throat, and flu, which ranged between 42.0% and 49.9% (p < 0.001). Nonetheless, it is important to note that none of the 16 different minor illnesses had a resolution rate below 40% (Table 6). The overall resolution rate observed during the second year was significantly higher than that of the first year (63.1% vs 61.8, respectively, p < 0.0001) (Table 6). When rates of resolution between the first and second year were compared for each of the 16 minor illnesses, it was found that resolution rates increased significantly in 8 (burns, emergency contraception, acute diarrhea, tooth pain, sore throat, lower urinary symptoms, pink eye and upper respiratory symptoms), decreased significantly in 5, and did not change significantly in the remaining 3 (Table 6). When patients were categorized in two groups according to age (using an arbitrary cutoff of 50 years), there were significant differences in the resolution rates of some categories; however, the overall resolution rate was identical in the two groups, indicating that age by itself has no major impact on the resolution rate in a program of nurse algorithm-guided care for adult patients (Table 7).

**Table 6:** Resolution rates of the 16 acute minor illnesess during the first and second year of the program

	**Resolution (%)**			
**Category**	**First year**	**Second year**	**Total**	p*
Burns	93.5	94.8	94.2	<0.0001
Emergency contraception	90.8	92.8	91.7	<0.0001
Skin injury	91.6	90.9	91.2	<0.0001
Skin fold dermatitis	71.3	69.3	70.3	0.017
Increased arterial pressure	70.0	67.2	68.4	<0.0001
Twisted ankle	66.0	66.6	66.3	NS
Nose bleeding	64.1	65.1	64.6	NS
Acute diarrhea	62.1	63.5	62.8	<0.0001
Tooth pain	54.1	56.1	55.2	<0.0001
Anxiety attacks	55.3	53.1	54.1	0.0013
Low back pain	51.4	51.2	51.6	NS
Flu	51.1	47.9	49.9	0.0026
Sore throat	45.7	49.9	47.8	<0.0001
Lower urinary symptoms	46.8	47.5	47.2	0.018
Pink eye	45.5	46.7	46.1	0.009
Upper respiratory symptoms	41.4	42.7	42	<0.0001
**Total**	**61.8**	**63.1**	**62.5**	<0.0001

*Comparison between the first and second year rates.

**Table 7:** Comparison of resolution rate of the different 16 minor acute illness in patients included categorized according to age*

	**Resolution by age (%)**		
**Category**	**≤ 50 (n = 751,325)**	>**50 (n = 458, 344)**	**p**
Burns	94.0	94.7	NS
Emergency contraception	-	-	-
Skin injury	89.7	92.0	<0.0001
Skin fold dermatitis	67.6	70.4	NS
Increased arterial pressure	61.1	68.7	<0.0001
Twisted ankle	65.6	65	NS
Nose bleeding	68.6	60.1	0.0046
Acute diarrhea	64.5	55.4	<0.0001
Tooth pain	54.4	57.1	0.0221
Anxiety attacks	50.3	60.3	<0.0001
Low back pain	49.7	52.5	<0.0001
Flu	50.7	45.3	0.0048
Sore throat	48.5	49.3	NS
Lower urinary symptoms	47.0	45.9	<0.0001
Pink eye	45.3	47.2	0.0103
Upper respiratory symptoms	44.8	37.4	<0.0001
**Total**	**60.1**	**60.1**	

*Emergency contraception was not included in the analysis because number of patients in the > 50 group was negligible.

In the whole series, the proportion of patients who returned to consultation after the nurse visit was low and averaged only 4.6%. The highest return rate was observed in skin injury and the lowest in emergency contraception (8.6% and 0.4%, respectively) (Table 8). The return to consultation of all 16 minor illnesses together was significantly higher during the second year compared to that of the first year (5.1% vs 4.2%, p < 0.0001) (Table 8). However, this increase in the return to consultation was basically due to the increment in burns and skin injury rates.

**Table 8:** Return to consultation of the 16 acute minor illness during the first and second year of the program

	**Return to consultation (%)**			
**Category**	**First year**	**Second year**	**Total**	p*
Burns	7.2	9.3	8.3	<0.0001
Emergency contraception	0.4	0.3	0.4	NS
Skin injury	7.5	9.6	8.6	<0.0001
Skin fold dermatitis	1.4	0.5	1.0	<0.0001
Increased arterial pressure	2.4	3.0	4.9	0.0006
Twisted ankle	1.5	1.9	1.7	0.0182
Nose bleeding	4.0	3.5	3.8	NS
Acute diarrhea	1.3	1.0	1.1	0.008
Tooth pain	1.9	2.1	2.0	NS
Anxiety attacks	1.4	1.1	1.3	NS
Low back pain	5.4	4.7	5.0	0.0008
Flu	1.0	0.7	0.8	NS
Sore throat	1.3	1.1	1.2	0.0239
Lower urinary symptoms	1.5	1.7	1.6	NS
Pink eye	1.3	1.3	1.3	NS
Upper respiratory symptoms	1.9	1.7	1.8	0.0063
**Total**	**4.2**	**5.1**	**4.6**	<0.0001

*Comparison between the first and second year rates.

## Discussion

In the current study we report the results of a program of nurse algorithm-guided care for adult patients with acute minor illnesses in primary care in a very large cohort of patients (1,209,669 consultations) over a two-year period. The program was designed to fulfill two criteria: treatment algorithms and close collaboration between nurses and GPs. The program was also intended to solve some of the concerns raised about the effectiveness and appropriateness of extending the role of nurses to same day consultations for acute minor illnesses in primary care, thus substituting GPs in this function. One of these concerns is that nurses may work independently of GPs which may result in lack of consultation in the case of uncertainty [13]. To avoid this potential effect, the program reported herein was the result of a collective effort between GPs and nurses. Moreover, in all management algorithms a number of signs of alarm were included, so that patients had to be sent to the GP for an urgent consultation if one of the signs of alarm was present. Another concern is that patients, at least in some countries, are not convinced that nurses have the sufficient education and knowledge to deal with these type of problems compared to GPs [11-14]. The close cooperation between nurses and GPs put forward in our program may help convince patients that nurses have the sufficient expertise to solve the problems in the majority of cases and that a consultation with the GP will be requested in case of uncertainty. A major issue that has limited the applicability of programs of nurse same day consultation for patients with acute minor illnesses in many countries has been the fact that nurses cannot prescribe certain drugs. On the other hand, nurses may have the feeling of a lack of appropriate pharmacological knowledge together with insufficient confidence in this area. The use of management algorithms allows restricting the drug prescription to certain conditions, following evidence-based guidelines. Moreover, the use of management algorithms may have the additional benefits of improving the adherence to protocols and reducing inter-professional variability in their application. Finally, the incorporation of the management algorithms in the computerized health record system is essential for their accurate application and also allows for a periodical assessment of the efficacy of the program.

The program reported here has some similarities with the walk-in centers from United Kingdom [15]. These are centers specifically created to provide care to patients with minor illnesses (minor injuries in many instances), which are usually run by nurses. However, there are significant differences that should be mentioned. First, our program was run in general practices and not in new and specific centers. Second, the program was devoted to a large number of acute minor illnesses that represent an important workload in primary care. Third, although the program was run by nurses, there was a close cooperation between nurses and GPs, which allowed the resolution of complex cases. Finally, the fact that the program was set at the general practice allowed a continuity of care.

Our results indicate that the rate of resolution achieved by nurses in 16 different minor illnesses is high (62.5%), with low probability of return to consultation for the same reason (below 5%). Interestingly, case resolution during the second year was higher compared to that of the first year, indicating that greater experience with the management algorithms resulted in an improved case resolution. We observed marked differences in the rate of case resolution between different conditions. For example, case resolution for burns and skin injury were much higher (over 90%) than those for lower urinary symptoms and upper respiratory symptoms, which did not reach 50%. Not resolved cases were sent to the GP for urgent consultation. Differences between resolution rates of the various minor illnesses might be related to the fact that some conditions correspond to long-established nurse practice (i.e. skin injury, burns) while others do not (i.e. upper respiratory symptoms, lower urinary symptoms). The relatively low resolution rate for these latter conditions probably indicates the compliance with signs of alarm that prompted referral to the GP as well as a high sense of responsibility of nurses in not assuming too complex processes. The rates of effectiveness in case resolution observed in the current study are high and support an approach using management algorithms for acute minor illnesses in primary care.

A limitation of the study is that case resolution was defined whenever the treatment protocol could be completed and treatment prescribed without the need of referral of the patient to the GP. Therefore, the resolution of the symptoms was not confirmed directly with the patient. Direct confirmation with the patient is hardly possible in a “real life” scenario. Return to consultation to primary care, either with a nurse or GP, was very low, averaging only 4.6%, which is keeping with the high rate of case resolution observed. It is important to note, however, that some patients could have requested a second consultation outside primary care and could have not been captured by the system. It is important to mention that patients’ satisfaction was not assessed in the current study. Therefore, the degree of satisfaction of patients by having been visited by a nurse instead of a GP could not be estimated. Randomized studies have demonstrated that in patients requesting same day consultation in primary care, the degree of satisfaction is greater when patients are treated by nurses than when they are treated by GPs [4,6,7,16]. There are however other studies with discrepant findings [11]. Finally, we did not evaluate the impact of the current approach in terms of cost. Further research will be required to evaluate the cost-effectiveness of the approach presented in this study.

## Conclusions

In conclusion, the current study reports the results of a program of nurse algorithm-guided care for adult patients with acute minor illnesses requesting same day consultation to primary care based on close cooperation between nurses and GPs. The application of this program proved to be very effective in case resolution. The use of this type of programs may help nurses expand their role in primary care.

## Competing interests

The authors declare that they have no competing interests.

## Authors’ contributions

NF conceived the study and participated in its design, analysis and interpretation of data, and writing of the manuscript. CS participated in the design of the study and analysis of data. EJ participated in the design of the study, analysis and interpretation of data, and helped to draft the manuscript. EA participated in the collection and analysis of data. DM participated in the collection and analysis of data. EC participated in the interpretation of data and helped to draft the manuscript. MU participated in the collection and analysis of data and helped to draft the manuscript. All authors have participated in the critical revision of the manuscript for important intellectual content and have read and approved the final manuscript.

## Pre-publication history

The pre-publication history for this paper can be accessed here:

http://www.biomedcentral.com/1471-2296/14/61/prepub
